# Supplementary material for: A Novel and Efficient Phthalate Hydrolase from Acinetobacter sp. LUNF3: Molecular Cloning, Characterization and Catalytic Mechanism
Source: Molecules. 2023 Sep 21;28(18):6738. doi: 10.3390/molecules28186738 (PMC10537300; doi:10.3390/molecules28186738)

Supplementary Materials for

A novel and efficient phthalate hydrolase from *Acinetobacter* sp. LUNF3: molecular cloning, characterization and catalytic mechanism

Shuanghu Fan [1,2,3], Jingjing Guo [4], Shaoyan Han [1], Haina Du [1], Zimeng Wang [1], Yajuan Fu [1,3], Hui Han [1,3], Xiaoqiang Hou [1,3,]* and Weixuan Wang [5,6,]*

[1] College of Life Science, Langfang Normal University, Langfang 065000, China; houxiaoqiang@lfnu.edu.cn, hanshaoyan2023@163.com, 18731308321@163.com, wzm20030512@163.com

[2] Institute of Agricultural Resources and Regional Planning, Chinese Academy of Agricultural Sciences, Beijing 100081, China; fanshuanghu@caas.cn

[3] Technical Innovation Center for Utilization of Edible and Medicinal Fungi in Hebei Province, Langfang 065000, China; fuyajuan501@163.com, hh071@163.com

[4] School of Chemistry and Materials Science, Langfang Normal University, Langfang 065000, China; guojingjing430@163.com

[5] Biotechnology Research Institute, Chinese Academy of Agricultural Sciences, Beijing 100081, China; wangweixuan@caas.cn

[6] National Nanfan Research Institute (Sanya), Chinese Academy of Agricultural Sciences, Sanya 572024, China

* Correspondence: houxiaoqiang@lfnu.edu.cn (X. H.); wangweixuan@caas.cn (W. W.)

Table S1. The top 100 sequences similar to the 16S sequence of strain LUNF3

Description	Scientific Name	Max Score	Total Score	Query Cover	E value	Per. ident	Acc. Len	Accession
Acinetobacter sp. strain LUNF3 16S ribosomal RNA gene, partial sequence	Acinetobacter sp.	2774	2774	100%	0	100	1502	OM900052.1
Acinetobacter sp. LUNF3 chromosome, complete genome	Acinetobacter sp. LUNF3	2756	####	100%	0	99.8	3317686	CP093968.1
Uncultured bacterium clone 5'-4 16S ribosomal RNA gene, partial sequence	uncultured bacterium	2752	2752	99%	0	99.8	1499	JQ923591.1
Uncultured Acinetobacter sp. clone 345 16S ribosomal RNA gene, partial sequence	uncultured Acinetobacter sp.	2750	2750	100%	0	99.7	1533	JN082588.1
Uncultured Acinetobacter sp. clone 171 16S ribosomal RNA gene, partial sequence	uncultured Acinetobacter sp.	2745	2745	100%	0	99.7	1533	JN082603.1
Uncultured Acinetobacter sp. clone 135 16S ribosomal RNA gene, partial sequence	uncultured Acinetobacter sp.	2745	2745	100%	0	99.7	1533	JN082546.1
Uncultured Acinetobacter sp. clone 344 16S ribosomal RNA gene, partial sequence	uncultured Acinetobacter sp.	2739	2739	100%	0	99.6	1533	JN082587.1
Uncultured Acinetobacter sp. clone 152 16S ribosomal RNA gene, partial sequence	uncultured Acinetobacter sp.	2739	2739	100%	0	99.6	1533	JN082553.1
Uncultured Acinetobacter sp. clone 138 16S ribosomal RNA gene, partial sequence	uncultured Acinetobacter sp.	2739	2739	100%	0	99.6	1533	JN082547.1
Uncultured Acinetobacter sp. clone 104 16S ribosomal RNA gene, partial sequence	uncultured Acinetobacter sp.	2739	2739	100%	0	99.6	1532	JN082541.1
Uncultured Acinetobacter sp. clone GI6-10b-E04 16S ribosomal RNA gene, partial sequence	uncultured Acinetobacter sp.	2739	2739	99%	0	99.7	1500	FJ193163.1
Uncultured Acinetobacter sp. clone GI6-10b-C06 16S ribosomal RNA gene, partial sequence	uncultured Acinetobacter sp.	2739	2739	99%	0	99.7	1501	FJ193150.1
Uncultured Acinetobacter sp. clone GI5-13-GI1 16S ribosomal RNA gene, partial sequence	uncultured Acinetobacter sp.	2737	2737	99%	0	99.6	1502	FJ192471.1

Uncultured bacterium clone SYN201307-7 16S ribosomal RNA gene, partial sequence	uncultured bacterium	2736	2736	99%	0	99.6	1499	KX508835.1
Uncultured Acinetobacter sp. clone XT20 16S ribosomal RNA gene, partial sequence	uncultured Acinetobacter sp.	2736	2736	99%	0	99.6	1499	KF511896.1
Uncultured Acinetobacter sp. clone GI5-007-B01 16S ribosomal RNA gene, partial sequence	uncultured Acinetobacter sp.	2736	2736	99%	0	99.7	1502	FJ192767.1
Uncultured Acinetobacter sp. clone GI5-13-E04 16S ribosomal RNA gene, partial sequence	uncultured Acinetobacter sp.	2736	2736	99%	0	99.7	1498	FJ192442.1
Uncultured Acinetobacter sp. clone GI5-13-B02 16S ribosomal RNA gene, partial sequence	uncultured Acinetobacter sp.	2736	2736	99%	0	99.6	1500	FJ192408.1
Acinetobacter johnsonii gene for 16S ribosomal RNA, partial sequence, strain: MTCC 9820	Acinetobacter johnsonii	2734	2734	100%	0	99.5	1502	AB859672.1
Uncultured Acinetobacter sp. clone 413 16S ribosomal RNA gene, partial sequence	uncultured Acinetobacter sp.	2734	2734	100%	0	99.5	1533	JN082595.1
Uncultured Acinetobacter sp. clone 397 16S ribosomal RNA gene, partial sequence	uncultured Acinetobacter sp.	2734	2734	100%	0	99.5	1533	JN082592.1
Uncultured Acinetobacter sp. clone 155 16S ribosomal RNA gene, partial sequence	uncultured Acinetobacter sp.	2734	2734	100%	0	99.5	1533	JN082554.1
Uncultured Acinetobacter sp. clone GI5-14-A04 16S ribosomal RNA gene, partial sequence	uncultured Acinetobacter sp.	2734	2734	99%	0	99.7	1493	FJ192483.1
Uncultured Acinetobacter sp. clone 314 16S ribosomal RNA gene, partial sequence	uncultured Acinetobacter sp.	2732	2732	100%	0	99.5	1531	JN082581.1
Uncultured Acinetobacter sp. clone GI5-13-C10 16S ribosomal RNA gene, partial sequence	uncultured Acinetobacter sp.	2732	2732	99%	0	99.7	1493	FJ192425.1
Uncultured bacterium clone ABH-04 16S ribosomal RNA gene, partial sequence	uncultured bacterium	2730	2730	99%	0	99.5	1499	KF964578.1
Uncultured bacterium clone B27 16S ribosomal RNA gene, partial sequence	uncultured bacterium	2730	2730	99%	0	99.5	1499	JN882063.1

Uncultured Acinetobacter sp. clone GI5-004-C05 16S ribosomal RNA gene, partial sequence	uncultured Acinetobacter sp.	2730	2730	99%	0	99.7	1495	FJ192622.1
Uncultured Acinetobacter sp. clone GI5-13-G03 16S ribosomal RNA gene, partial sequence	uncultured Acinetobacter sp.	2730	2730	99%	0	99.5	1498	FJ192464.1
Acinetobacter johnsonii strain M19 chromosome, complete genome	Acinetobacter johnsonii	2728	####	100%	0	99.5	3749210	CP037424.1
Uncultured Acinetobacter sp. gene for 16S ribosomal RNA, partial sequence, clone: 21d138	uncultured Acinetobacter sp.	2728	2728	99%	0	99.5	1498	LC140820.1
Acinetobacter johnsonii strain FDAARGOS_1092 chromosome, complete genome	Acinetobacter johnsonii	2728	####	100%	0	99.5	3604384	CP068206.1
Uncultured Acinetobacter sp. clone 369 16S ribosomal RNA gene, partial sequence	uncultured Acinetobacter sp.	2728	2728	100%	0	99.5	1532	JN082590.1
Uncultured Acinetobacter sp. clone 357 16S ribosomal RNA gene, partial sequence	uncultured Acinetobacter sp.	2728	2728	100%	0	99.5	1532	JN082589.1
Uncultured Acinetobacter sp. clone 303 16S ribosomal RNA gene, partial sequence	uncultured Acinetobacter sp.	2728	2728	100%	0	99.5	1532	JN082579.1
Uncultured Acinetobacter sp. clone 226 16S ribosomal RNA gene, partial sequence	uncultured Acinetobacter sp.	2728	2728	100%	0	99.5	1532	JN082570.1
Uncultured Acinetobacter sp. clone 92 16S ribosomal RNA gene, partial sequence	uncultured Acinetobacter sp.	2728	2728	100%	0	99.5	1533	JN082539.1
Uncultured Acinetobacter sp. clone 85 16S ribosomal RNA gene, partial sequence	uncultured Acinetobacter sp.	2728	2728	100%	0	99.5	1533	JN082538.1
Uncultured bacterium clone BXHA11 16S ribosomal RNA gene, partial sequence	uncultured bacterium	2728	2728	99%	0	99.5	1498	GQ479969.1
Uncultured bacterium clone DQB-W153 16S ribosomal RNA gene, partial sequence	uncultured bacterium	2728	2728	100%	0	99.5	1503	GQ415368.1
Uncultured Acinetobacter sp. clone GI5-002-F12 16S ribosomal RNA gene, partial sequence	uncultured Acinetobacter sp.	2728	2728	99%	0	99.6	1497	FJ193020.1

Uncultured Acinetobacter sp. clone GI5-004-C06 16S ribosomal RNA gene, partial sequence	uncultured Acinetobacter sp.	2728	2728	99%	0	99.6	1495	FJ192623.1
Uncultured Acinetobacter sp. clone GI5-14-G07 16S ribosomal RNA gene, partial sequence	uncultured Acinetobacter sp.	2728	2728	99%	0	99.6	1496	FJ192535.1
Uncultured gamma proteobacterium BioIuz K32 16S ribosomal RNA gene, partial sequence	uncultured gamma proteobacterium BioIuz K32	2728	2728	100%	0	99.5	1503	AF324537.1
Uncultured Acinetobacter sp. clone GI5-007-F03 16S ribosomal RNA gene, partial sequence	uncultured Acinetobacter sp.	2726	2726	99%	0	99.6	1499	FJ192805.1
Uncultured Acinetobacter sp. clone GI5-14-F11 16S ribosomal RNA gene, partial sequence	uncultured Acinetobacter sp.	2726	2726	99%	0	99.6	1497	FJ192529.1
Uncultured Acinetobacter sp. clone GI5-14-C04 16S ribosomal RNA gene, partial sequence	uncultured Acinetobacter sp.	2726	2726	99%	0	99.7	1489	FJ192494.1
Acinetobacter johnsonii partial 16S rRNA gene, strain HAMBI 1969	Acinetobacter johnsonii	2724	2724	99%	0	99.5	1513	LT899949.1
Uncultured Acinetobacter sp. clone b12-71 16S ribosomal RNA gene, partial sequence	uncultured Acinetobacter sp.	2724	2724	99%	0	99.5	1505	JX575959.1
Uncultured bacterium clone 5'-105 16S ribosomal RNA gene, partial sequence	uncultured bacterium	2724	2724	99%	0	99.5	1499	JQ923658.1
Uncultured bacterium clone 5'-31 16S ribosomal RNA gene, partial sequence	uncultured bacterium	2724	2724	99%	0	99.5	1499	JQ923614.1
Uncultured Acinetobacter sp. clone GI6-10b-B05 16S ribosomal RNA gene, partial sequence	uncultured Acinetobacter sp.	2724	2724	99%	0	99.5	1496	FJ193142.1
Uncultured Acinetobacter sp. clone GI5-007-H09 16S ribosomal RNA gene, partial sequence	uncultured Acinetobacter sp.	2724	2724	99%	0	99.6	1494	FJ192831.1
Uncultured Acinetobacter sp. clone GI5-007-C02 16S ribosomal RNA gene, partial sequence	uncultured Acinetobacter sp.	2724	2724	99%	0	99.7	1494	FJ192779.1

Uncultured Acinetobacter sp. clone GI5-13-B10 16S ribosomal RNA gene, partial sequence	uncultured Acinetobacter sp.	2724	2724	99%	0	99.5	1498	FJ192415.1
Acinetobacter sp. D1103 16S ribosomal RNA gene, partial sequence	Acinetobacter sp. D1103	2724	2724	99%	0	99.5	1496	DQ480137.1
Uncultured bacterium clone EV821FW101601SAC11 16S ribosomal RNA gene, partial sequence	uncultured bacterium	2724	2724	99%	0	99.5	1499	DQ226081.1
Acinetobacter sp. NEB 394 chromosome, complete genome	Acinetobacter sp. NEB 394	2723	####	100%	0	99.4	3479933	CP055277.1
Acinetobacter johnsonii strain M19 16S ribosomal RNA gene, partial sequence	Acinetobacter johnsonii	2723	2723	100%	0	99.4	1526	MT226917.1
Acinetobacter johnsonii strain LXL_C1 chromosome, complete genome	Acinetobacter johnsonii	2723	####	100%	0	99.4	3398706	CP031011.1
Acinetobacter johnsonii strain ICE_NC chromosome, complete genome	Acinetobacter johnsonii	2723	####	100%	0	99.4	3672417	CP090416.1
Acinetobacter johnsonii strain ATCC 17909 16S ribosomal RNA, partial sequence	Acinetobacter johnsonii	2723	2723	100%	0	99.4	1530	NR_117624.1
Acinetobacter johnsonii strain FDAARGOS_1093 chromosome, complete genome	Acinetobacter johnsonii	2723	####	100%	0	99.4	3633703	CP068195.1
Acinetobacter johnsonii strain FDAARGOS_910 chromosome, complete genome	Acinetobacter johnsonii	2723	####	100%	0	99.4	3598417	CP065666.1
Uncultured bacterium clone SupB1022 16S ribosomal RNA gene, partial sequence	uncultured bacterium	2723	2723	100%	0	99.4	1534	MW127756.1
Uncultured Acinetobacter sp. clone GI5-002-B06 16S ribosomal RNA gene, partial sequence	uncultured Acinetobacter sp.	2723	2723	99%	0	99.5	1501	FJ192980.1
Uncultured Acinetobacter sp. clone GI5-004-F05 16S ribosomal RNA gene, partial sequence	uncultured Acinetobacter sp.	2723	2723	99%	0	99.5	1495	FJ192653.1

Uncultured Acinetobacter sp. clone GI5-008-H11 16S ribosomal RNA gene, partial sequence	uncultured Acinetobacter sp.	2723	2723	99%	0	99.5	1499	FJ192601.1
Uncultured Acinetobacter sp. clone GI5-13-E02 16S ribosomal RNA gene, partial sequence	uncultured Acinetobacter sp.	2723	2723	99%	0	99.5	1496	FJ192440.1
Uncultured Acinetobacter sp. clone GI5-13-C03 16S ribosomal RNA gene, partial sequence	uncultured Acinetobacter sp.	2723	2723	99%	0	99.5	1495	FJ192418.1
Acinetobacter sp. mixed culture X12-17 16S ribosomal RNA gene, partial sequence	Acinetobacter sp. mixed culture X12-17	2721	2721	99%	0	99.4	1500	KR029401.1
Uncultured Acinetobacter sp. clone GI5-14-E05 16S ribosomal RNA gene, partial sequence	uncultured Acinetobacter sp.	2721	2721	99%	0	99.5	1498	FJ192513.1
Uncultured Acinetobacter sp. clone GI5-13-C04 16S ribosomal RNA gene, partial sequence	uncultured Acinetobacter sp.	2721	2721	99%	0	99.5	1495	FJ192419.1
Uncultured bacterium clone MP104-1109-b35 16S ribosomal RNA gene, partial sequence	uncultured bacterium	2721	2721	99%	0	99.4	1500	DQ088799.1
Acinetobacter sp. strain SYSU_D00866 16S ribosomal RNA gene, partial sequence	Acinetobacter sp.	2719	2719	99%	0	99.4	1499	MT527668.1
Uncultured bacterium clone OTU44 16S ribosomal RNA gene, partial sequence	uncultured bacterium	2719	2719	99%	0	99.5	1496	KP975300.1
Uncultured Acinetobacter sp. gene for 16S ribosomal RNA, partial sequence, clone: 3CP(+)2_60	uncultured Acinetobacter sp.	2719	2719	99%	0	99.5	1499	AB908751.1
Uncultured bacterium clone XXM_1_017 16S ribosomal RNA gene, partial sequence	uncultured bacterium	2719	2719	99%	0	99.5	1493	JX559164.1
Uncultured bacterium clone 6B-17 16S ribosomal RNA gene, partial sequence	uncultured bacterium	2719	2719	99%	0	99.4	1499	JQ923747.1
Uncultured Acinetobacter sp. partial 16S rRNA gene, isolate ELC0606, clone A06T08C05	uncultured Acinetobacter sp.	2719	2719	99%	0	99.4	1501	HE575541.1

Uncultured marine bacterium clone BM1-4-12 16S ribosomal RNA gene, partial sequence	uncultured marine bacterium	2719	2719	99%	0	99.5	1492	FJ825890.1
Uncultured bacterium clone KZNMV-30-B70 16S ribosomal RNA gene, partial sequence	uncultured bacterium	2719	2719	99%	0	99.5	1496	FJ712619.1
Uncultured Acinetobacter sp. clone GI5-005-G05 16S ribosomal RNA gene, partial sequence	uncultured Acinetobacter sp.	2719	2719	98%	0	99.7	1485	FJ192743.1
Uncultured Acinetobacter sp. clone GI5-004-G09 16S ribosomal RNA gene, partial sequence	uncultured Acinetobacter sp.	2719	2719	98%	0	99.7	1495	FJ192669.1
Uncultured Acinetobacter sp. clone GI5-13-F07 16S ribosomal RNA gene, partial sequence	uncultured Acinetobacter sp.	2719	2719	99%	0	99.5	1494	FJ192457.1
uncultured bacterium clone BotBa17 16S ribosomal RNA gene, partial sequence	uncultured bacterium	2719	2719	99%	0	99.4	1500	EF999405.1
Uncultured bacterium clone EV818BHEB5102702SAS12 16S ribosomal RNA gene, partial sequence	uncultured bacterium	2719	2719	99%	0	99.4	1499	DQ256340.1
Acinetobacter johnsonii strain ATCC 17909 16S ribosomal RNA gene, partial sequence	Acinetobacter johnsonii	2717	2717	100%	0	99.3	1529	MK184297.1
Acinetobacter johnsonii strain IC001 chromosome, complete genome	Acinetobacter johnsonii	2717	####	100%	0	99.3	3610322	CP022298.1
Uncultured bacterium clone HJ-32 16S ribosomal RNA gene, partial sequence	uncultured bacterium	2717	2717	99%	0	99.4	1498	KJ643964.1
Acinetobacter johnsonii strain AYTCM chromosome, complete genome	Acinetobacter johnsonii	2717	####	100%	0	99.3	3567832	CP121776.1
Acinetobacter johnsonii XBB1, complete genome	Acinetobacter johnsonii XBB1	2717	####	100%	0	99.3	3509795	CP010350.1
Acinetobacter johnsonii strain AJ413 chromosome, complete genome	Acinetobacter johnsonii	2717	####	100%	0	99.3	3740853	CP045103.1
Uncultured Acinetobacter sp. clone DM4-30 16S ribosomal RNA gene, partial sequence	uncultured Acinetobacter sp.	2717	2717	99%	0	99.4	1498	KC172242.1

Acinetobacter johnsonii strain E10B chromosome, complete genome	Acinetobacter johnsonii	2717	####	100%	0	99.3	3521987	CP059080.1
Uncultured bacterium clone HWGB-46 16S ribosomal RNA gene, partial sequence	uncultured bacterium	2717	2717	100%	0	99.3	1503	JQ684304.1
Uncultured Acinetobacter sp. clone 222 16S ribosomal RNA gene, partial sequence	uncultured Acinetobacter sp.	2717	2717	100%	0	99.3	1532	JN082566.1
Uncultured Acinetobacter sp. clone 219 16S ribosomal RNA gene, partial sequence	uncultured Acinetobacter sp.	2717	2717	100%	0	99.3	1532	JN082564.1
Uncultured Acinetobacter sp. clone 195 16S ribosomal RNA gene, partial sequence	uncultured Acinetobacter sp.	2717	2717	100%	0	99.3	1532	JN082557.1
Uncultured Acinetobacter sp. clone GI5-004-E09 16S ribosomal RNA gene, partial sequence	uncultured Acinetobacter sp.	2715	2715	98%	0	99.7	1489	FJ192645.1

Table S2. The genomic characteristics of *Acinetobacter* sp. LUNF3.

**Features**	**Chromosome**	**Plasmid**
Length (bp)	3,317,686	101,829
GC content (%)	41.43	38.14
Protein-coding sequences (CDSs)	3177	98
rRNA (5S, 16S, 23S)	21(7, 7,7)	0
tRNA	89	0
ncRNA	0	1
CRISPRs	1	0
CDSs annotated in NR	3,131	92
CDSs annotated in COG	2,688	71
CDSs annotated in KEGG	1,607	0
CDSs annotated in Swiss-Prot	2,121	41
CDSs annotated in GO	2,214	56

Table S3. The sequence of primers used for site-directed mutagenesis of DphAN1.

Primers	Sequences（5'-3'）
S201A-F	ATgccGCGGGTGGTAATATTAGCGCGGTTGTG
S201A-R	ATTACCACCCGCggcATCGCCCGCCACCGCAAT
D295A-F	ATgccCTGCTGCATGATGAAGGCGAAATTTAT
D295A-R	ATCATGCAGCAGggcATGACCCGCGGTAATAACAAAC
H325A-F	GgccGGCTTTGTGAATTTTACCATTATTAGCC
H325A-R	AATTCACAAAGCCggcCGGCTGATCCAGATAATTCTGAT
G129A-F	TGTTTTATCACGGCgccGGCTTTGTGGTGGGCAGC
G129A-R	ggcGCCGTGATAAAACACCAGCATCGGCAGTT
G130A-F	ATCACGGCGGCgccTTTGTGGTGGGCAGCTTAGATA
G130A-R	AAAggcGCCGCCGTGATAAAACACCAGCATCG
F78A-F	CAAACTGgccGATCATCAGATTCAGATGCTGACC
F78A-R	GATGATCggcCAGTTTGCGGCTGCGGTGATAG
V133A-F	TTTGTGgccGGCAGCTTAGATAGCCATGATGA
V133A-R	AAGCTGCCggcCACAAAGCCGCCGCCGTGATA
V230A-F	TTATCCGgccGTTGATTTTAAGAGCCGCCATC
V230A-R	AATCAACggcCGGATAAATCAGTAATTGCGCG
D253A-F	AGgccGTGGATCGTGTTACCAGCCTGTATGCG
D253A-R	AACACGATCCACggcCTGGCCGGTCAGGGTCAG
V254A-F	AGGATgccGATCGTGTTACCAGCCTGTATGCG
V254A-R	AACACGATCggcATCCTGGCCGGTCAGGGTCA
V257A-F	TGTGGATCGTgccACCAGCCTGTATGCGGAACA
V257A-R	TGGTggcACGATCCACATCCTGGCCGGTCAGG
F330A-F	CTTTGTGAATgccACCATTATTAGCCGCCGCG
F330A-R	TGGTggcATTCACAAAGCCATGCGGCTGATCC

Figure S1. The HPLC profile of DBP degraded by strain LUNF3 (B) compared with control (A). CK: TEM (pH8.0) with 0.5 mM DBP.

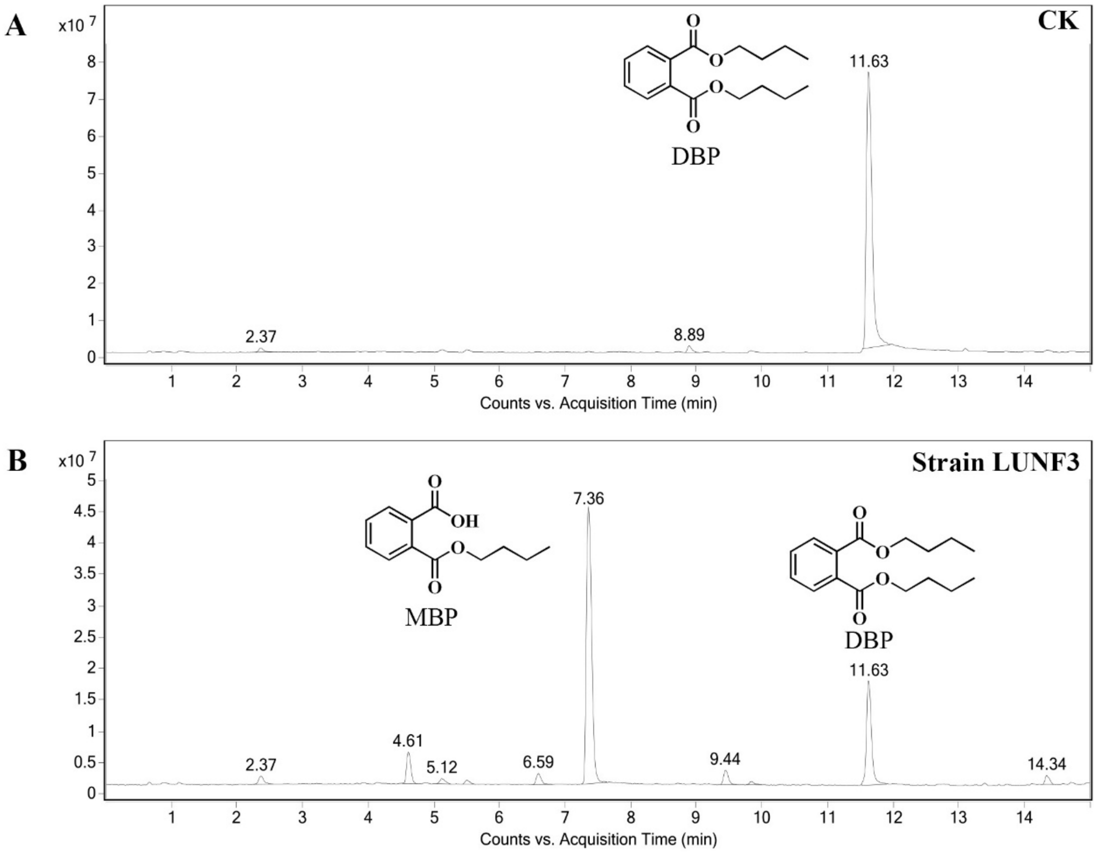

Figure S2. The substrates detection of strain LUNF3 (A) and metabolites detected by HPLC-MS (B) (* $p < 0.05$, ** $p < 0.01$ and *** $p < 0.0001$). CK (A): TEM (Ph8.0) with 0.5 Mm DEP, DBP or BBP. CK (B): TEM (pH8.0) with 0.5 mM DEP.

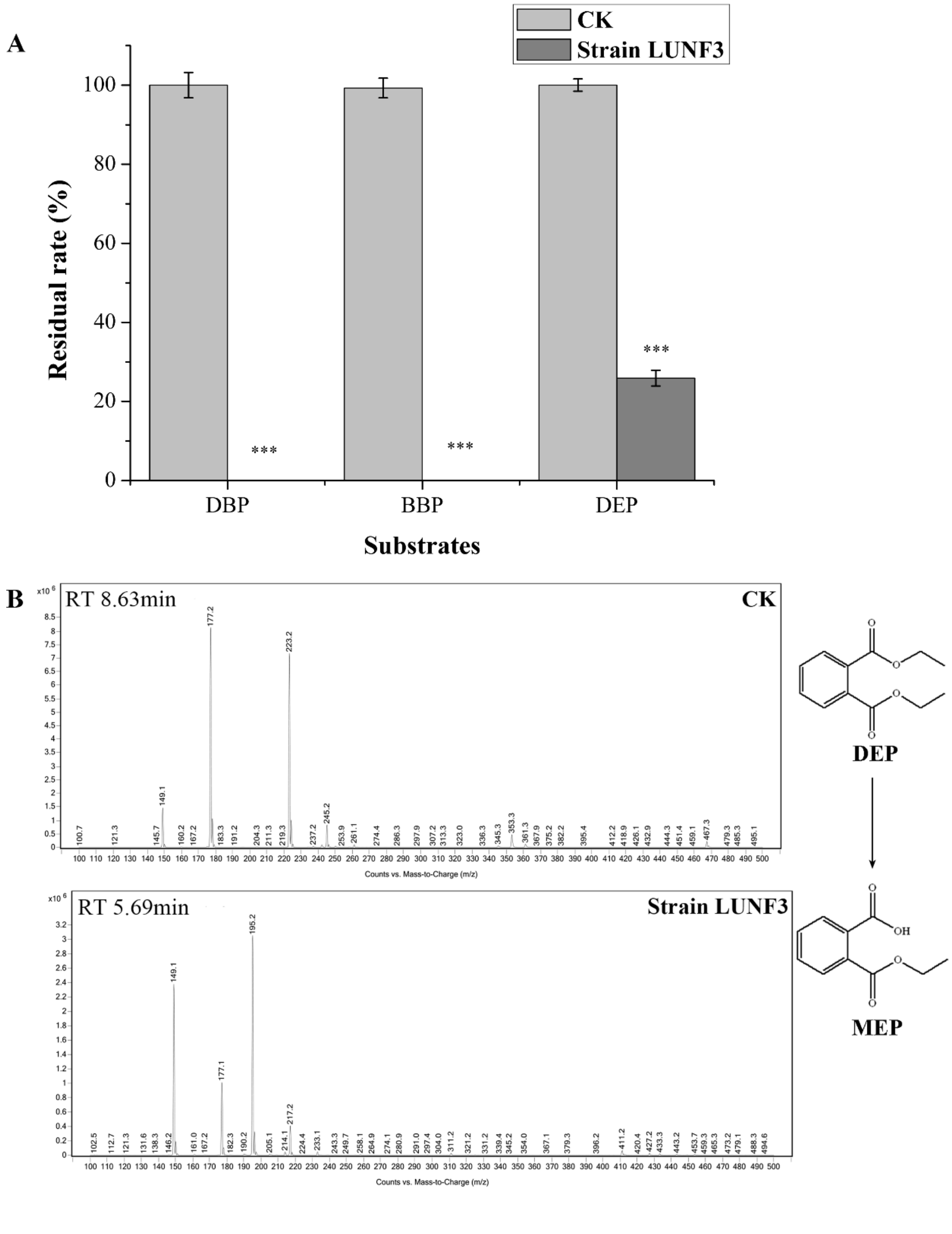

Figure S3. The genes from chromosome (A) and plasmid (B) of strain LUNF3 annotated in GO database and the distribution of these genes in metabolic pathways based on KEGG database (C).

A

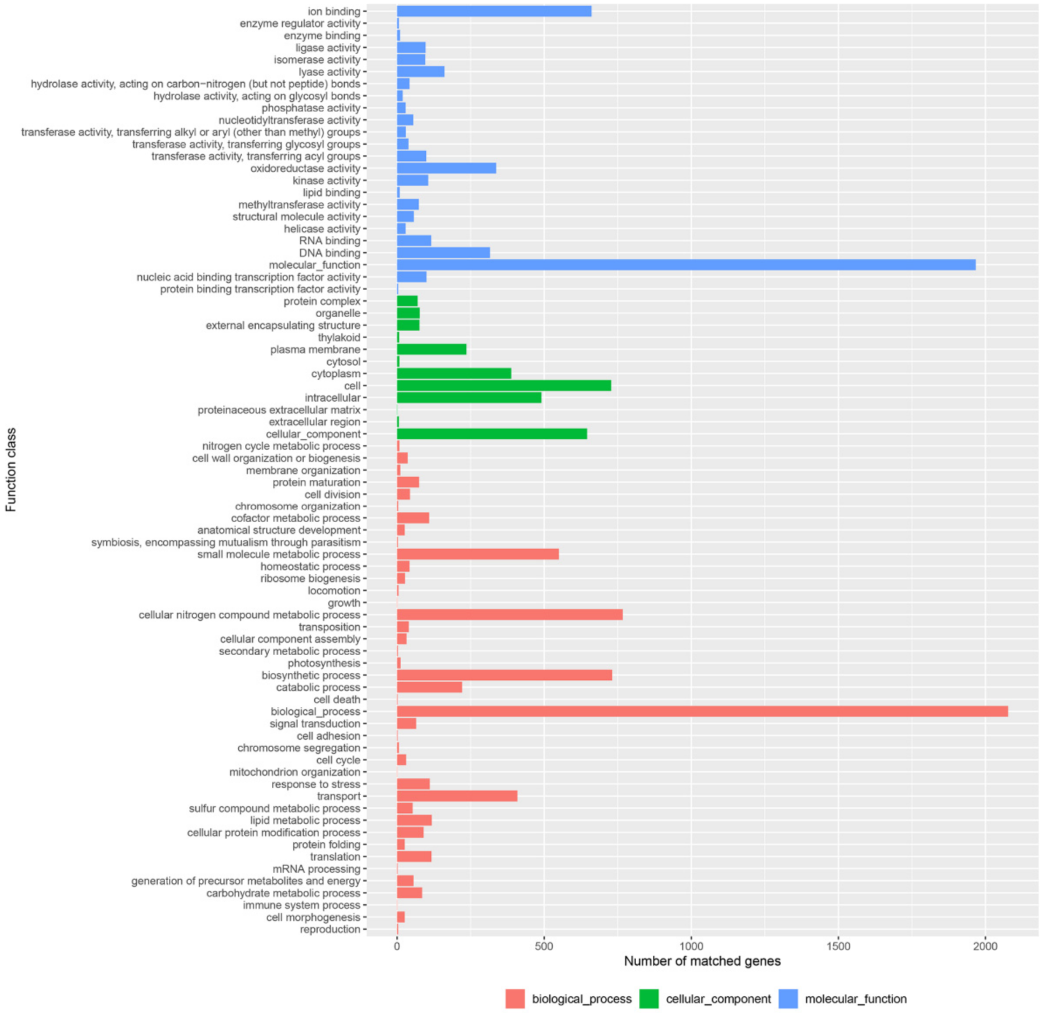

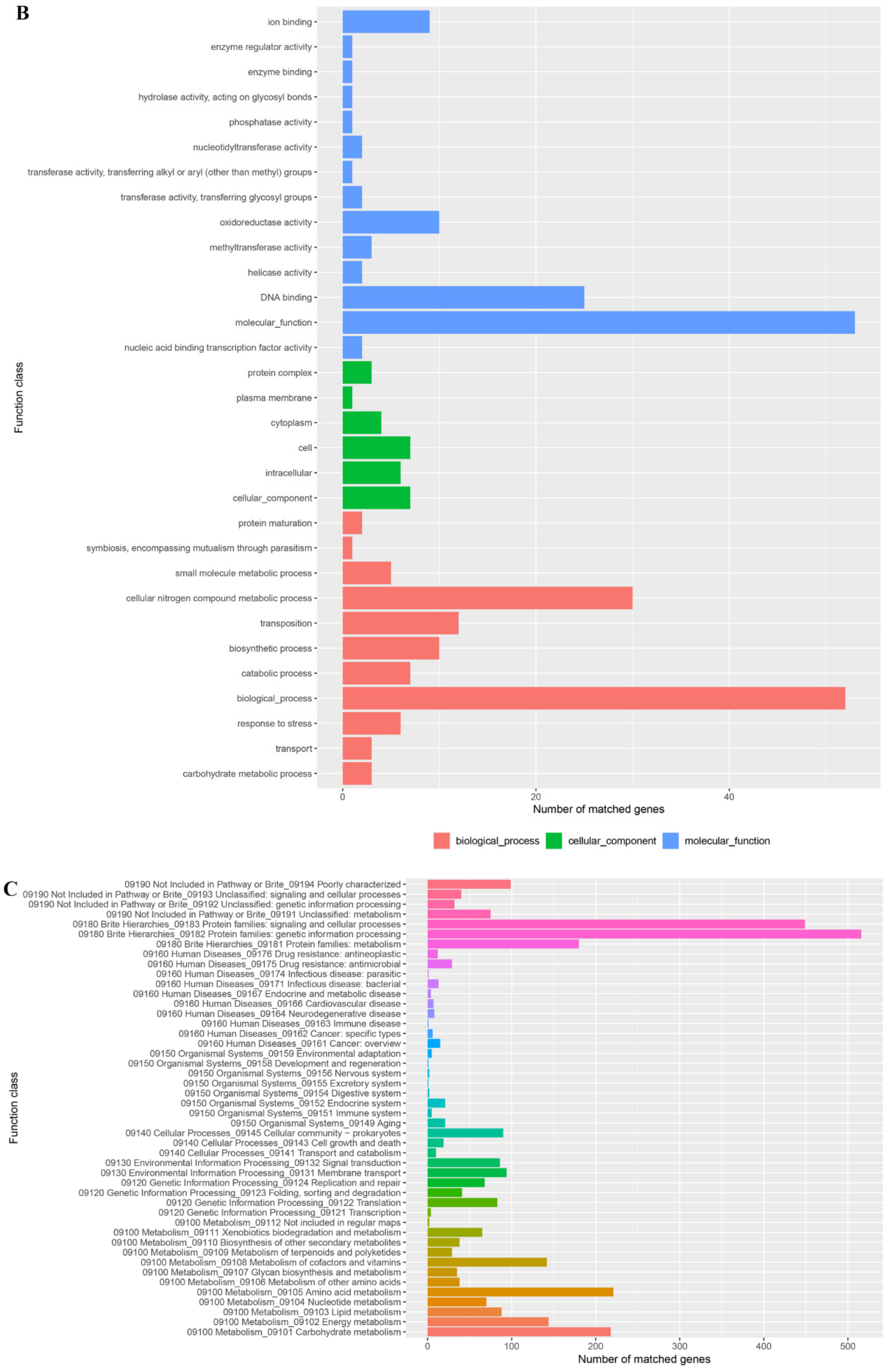
B
ion binding
enzyme regulator activity
enzyme binding
hydrolase activity, acting on glycosyl bonds
phosphatase activity
nucleotidyltransferase activity
transferase activity, transferring alkyl or aryl (other than methyl) groups
transferase activity, transferring glycosyl groups
oxidoreductase activity
methyltransferase activity
helicase activity
DNA binding
molecular_function
nucleic acid binding transcription factor activity
protein complex
plasma membrane
cytoplasm
cell
intracellular
cellular_component
protein maturation
symbiosis, encompassing mutualism through parasitism
small molecule metabolic process
cellular nitrogen compound metabolic process
transposition
biosynthetic process
catabolic process
biological_process
response to stress
transport
carbohydrate metabolic process
Function class
0
20
40
Number of matched genes
biological_process
cellular_component
molecular_function
C
09190 Not Included in Pathway or Brite_09194 Poorly characterized
09190 Not Included in Pathway or Brite_09193 Unclassified: signaling and cellular processes
09190 Not Included in Pathway or Brite_09192 Unclassified: genetic information processing
09190 Not Included in Pathway or Brite_09191 Unclassified: metabolism
09180 Brite Hierarchies_09183 Protein families: signaling and cellular processes
09180 Brite Hierarchies_09182 Protein families: genetic information processing
09180 Brite Hierarchies_09181 Protein families: metabolism
09160 Human Diseases_09176 Drug resistance: antineoplastic
09160 Human Diseases_09175 Drug resistance: antimicrobial
09160 Human Diseases_09174 Infectious disease: parasitic
09160 Human Diseases_09171 Infectious disease: bacterial
09160 Human Diseases_09167 Endocrine and metabolic disease
09160 Human Diseases_09166 Cardiovascular disease
09160 Human Diseases_09164 Neurodegenerative disease
09160 Human Diseases_09163 Immune disease
09160 Human Diseases_09162 Cancer: specific types
09160 Human Diseases_09161 Cancer: overview
09150 Organismal Systems_09159 Environmental adaptation
09150 Organismal Systems_09158 Development and regeneration
09150 Organismal Systems_09156 Nervous system
09150 Organismal Systems_09155 Excretory system
09150 Organismal Systems_09154 Digestive system
09150 Organismal Systems_09152 Endocrine system
09150 Organismal Systems_09151 Immune system
09150 Organismal Systems_09149 Aging
09140 Cellular Processes_09145 Cellular community − prokaryotes
09140 Cellular Processes_09143 Cell growth and death
09140 Cellular Processes_09141 Transport and catabolism
09130 Environmental Information Processing_09132 Signal transduction
09130 Environmental Information Processing_09131 Membrane transport
09120 Genetic Information Processing_09124 Replication and repair
09120 Genetic Information Processing_09123 Folding, sorting and degradation
09120 Genetic Information Processing_09122 Translation
09120 Genetic Information Processing_09121 Transcription
09100 Metabolism_09112 Not included in regular maps
09100 Metabolism_09111 Xenobiotics biodegradation and metabolism
09100 Metabolism_09110 Biosynthesis of other secondary metabolites
09100 Metabolism_09109 Metabolism of terpenoids and polyketides
09100 Metabolism_09108 Metabolism of cofactors and vitamins
09100 Metabolism_09107 Glycan biosynthesis and metabolism
09100 Metabolism_09106 Metabolism of other amino acids
09100 Metabolism_09105 Amino acid metabolism
09100 Metabolism_09104 Nucleotide metabolism
09100 Metabolism_09103 Lipid metabolism
09100 Metabolism_09102 Energy metabolism
09100 Metabolism_09101 Carbohydrate metabolism
Function class
0
100
200
300
400
500
Number of matched genes

Figure S4. The functional classification of genes from chromosome (A) and plasmid (B) based on COG database.

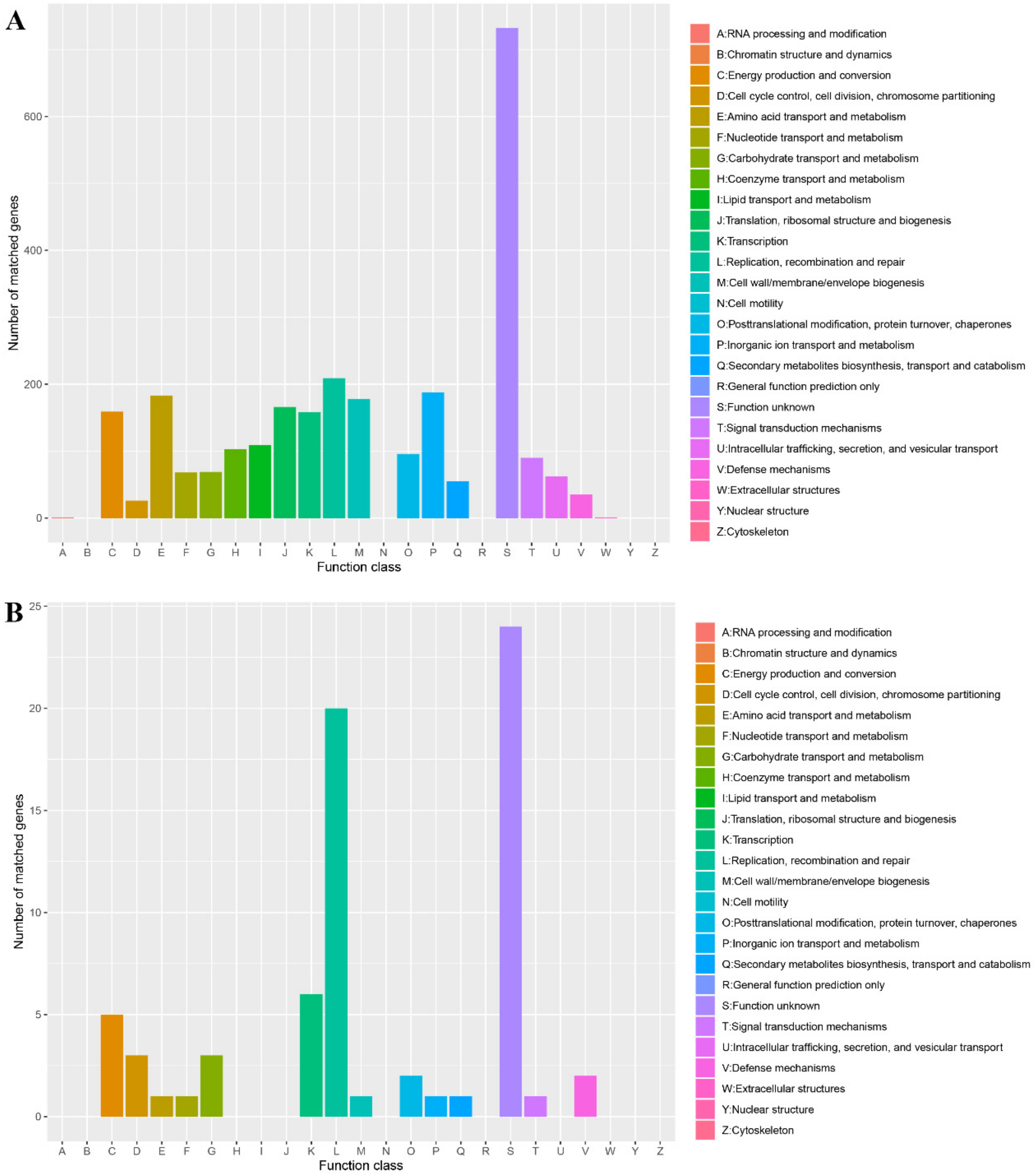

Figure S5. The alignment of DphAN1 and other hydrolases from family IV.

The conserved amino acid residues are shown in rectangle. The catalytic triad (Ser[201]-Asp[295]-His[325]) is marked by pentastar.

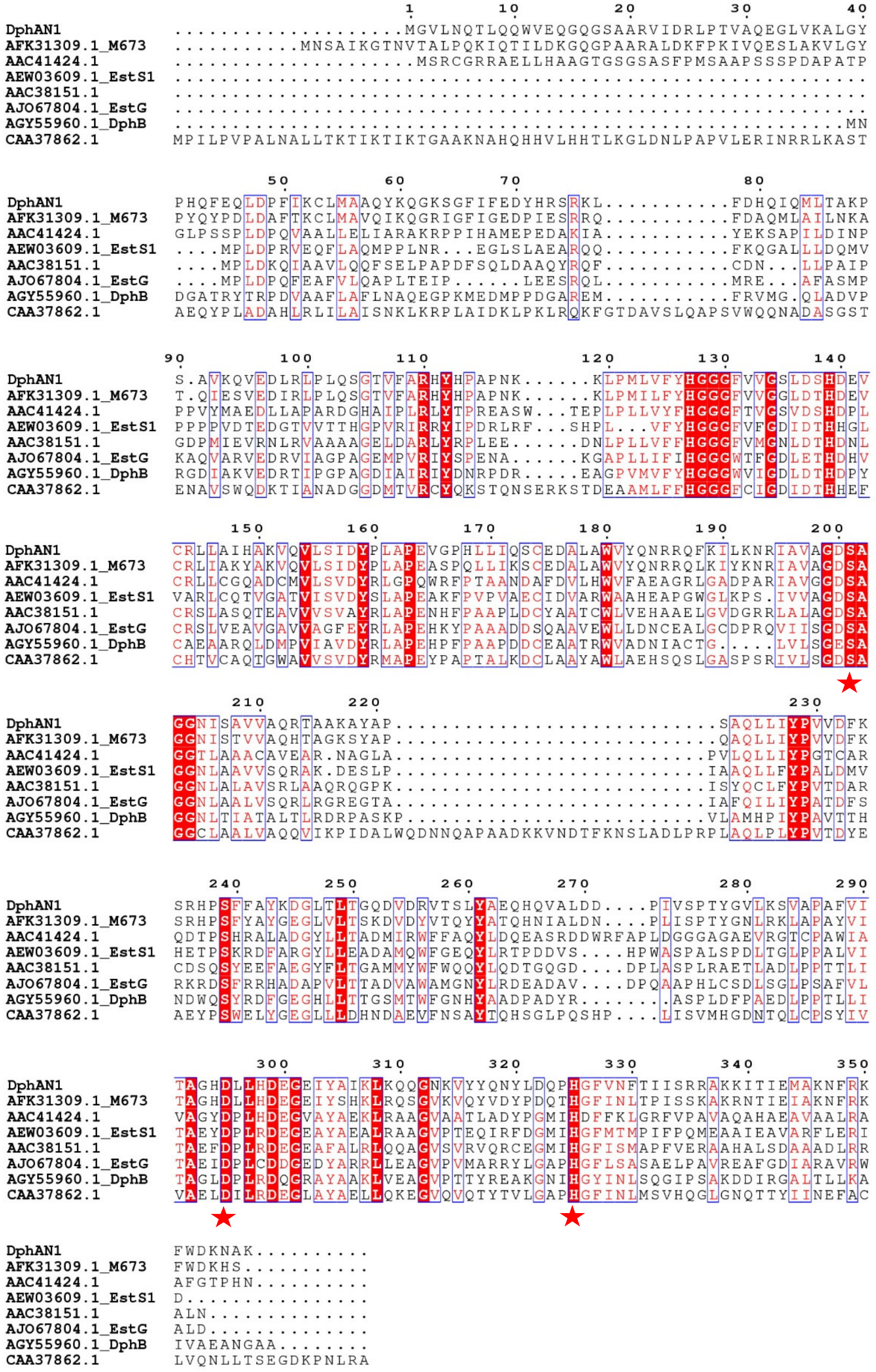

Figure S6. The highly conserved motifs $D^{295}LLHDEG^{301}$ (A), $H^{325}GF^{327}$ (B) and $V^{154}LSIDYPLAPE^{164}$ (C) detected from DphAN1.

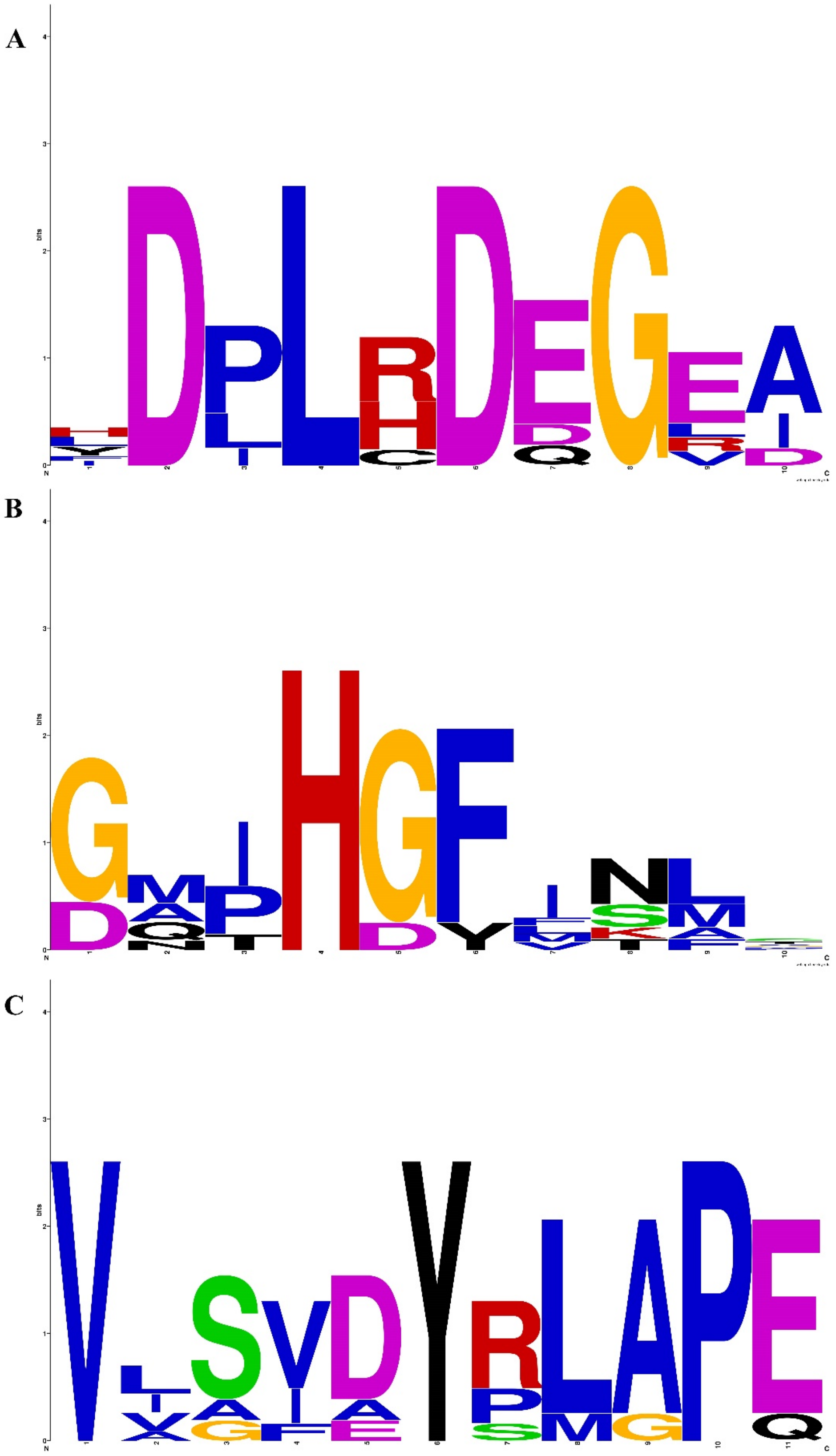

Figure S7. The HPLC-MS spectrum of DEP (A) and BBP (B) catalyzed by DphAN1. CK (A): Tris-HCl (50 mM, pH8.0) with 0.5 mM DEP. CK (B): Tris-HCl (50 mM, pH8.0) with 0.5 mM BBP.

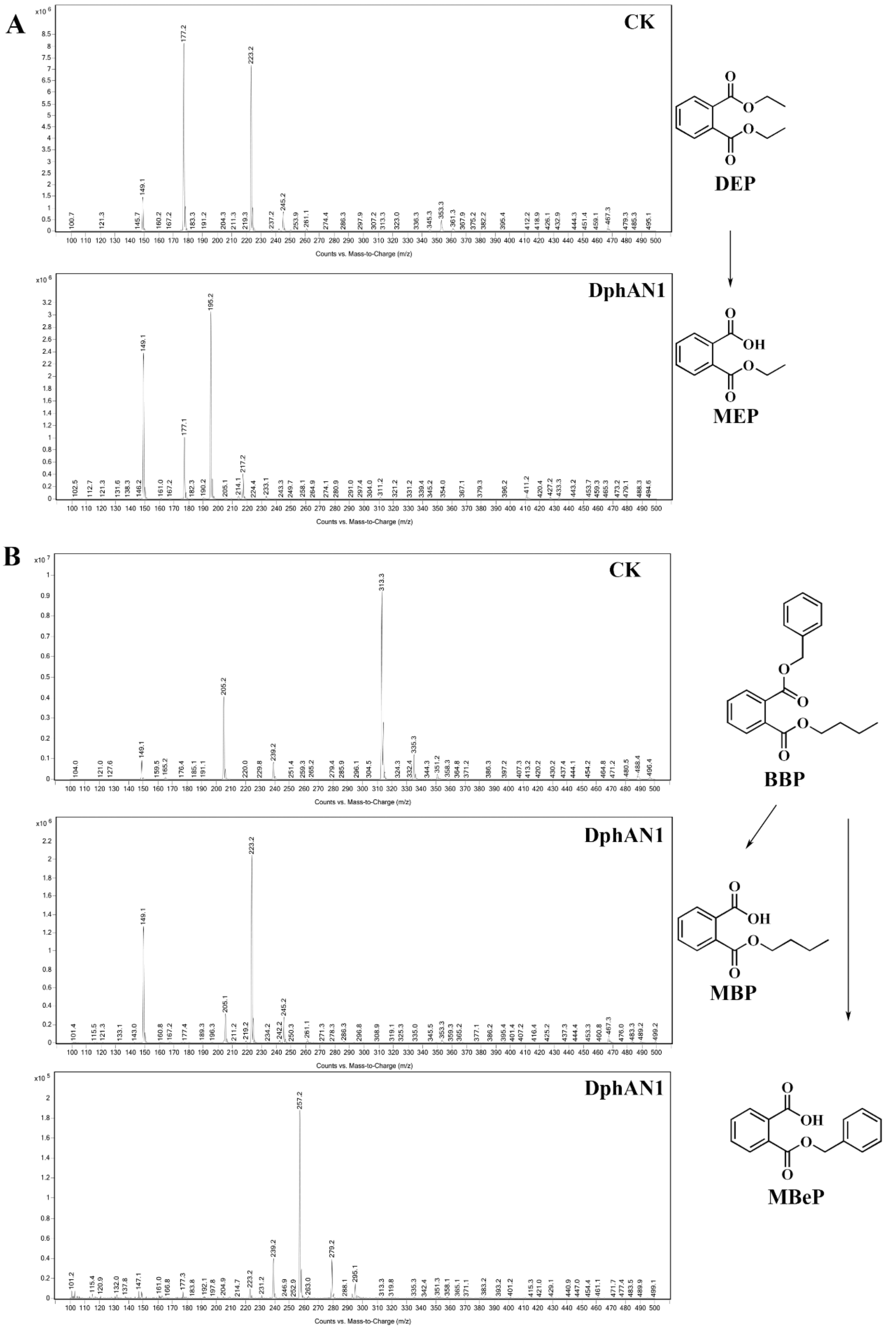

Figure S8. The evaluation of the model structure of DphAN1 by MolProbity Ramachandran analysis.

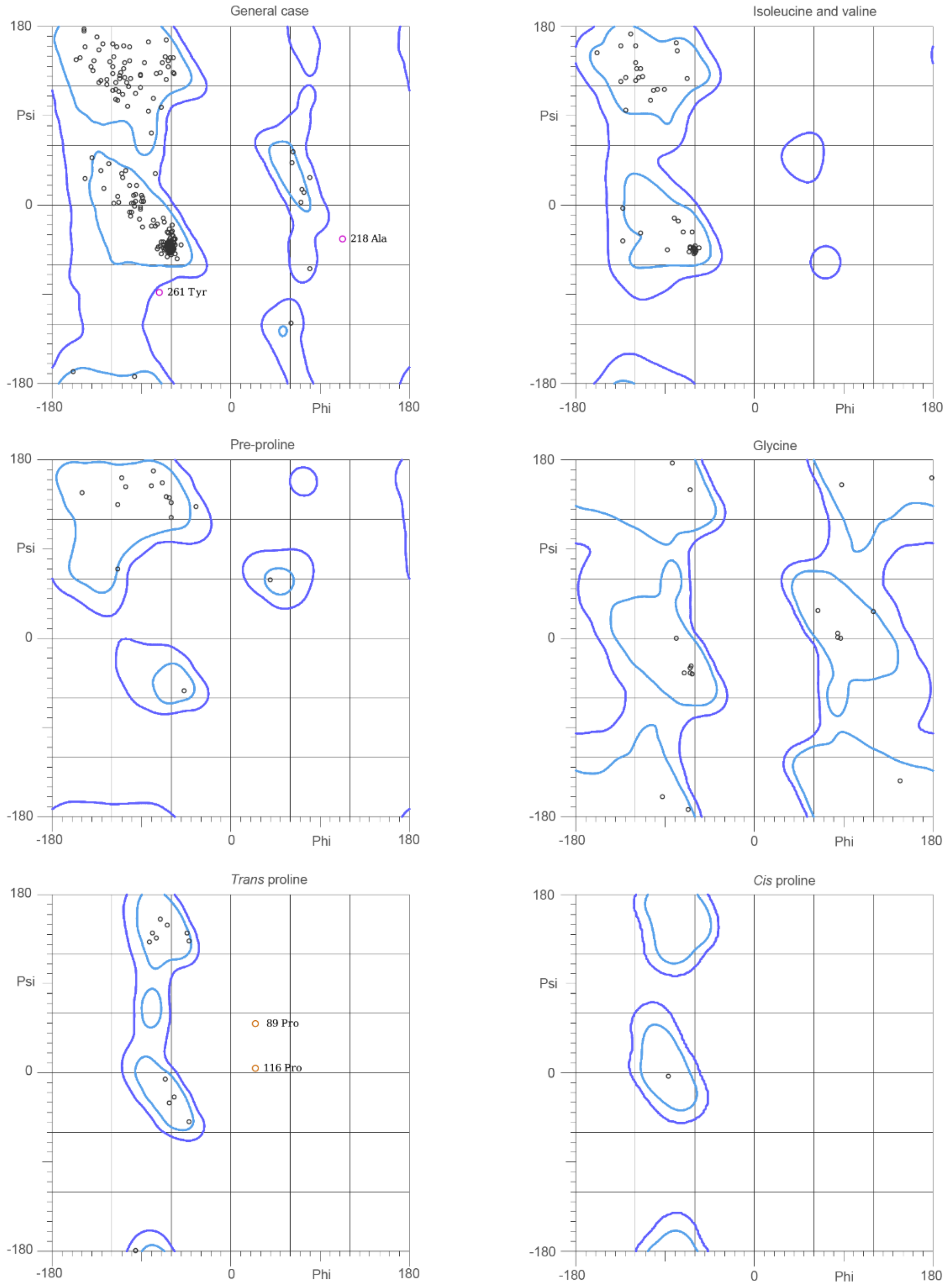

94.8% (292/308) of all residues were in favored (98%) regions.
98.7% (304/308) of all residues were in allowed (>99.8%) regions.

There were 4 outliers (phi, psi):
89 Pro (25.4, 50.1)
116 Pro (25.8, 5.2)
218 Ala (114.0, -34.4)
261 Tyr (-72.3, -88.6)

Figure S9. The hydrophobic pocket of hydrolase DphAN1 where DBP was docked.

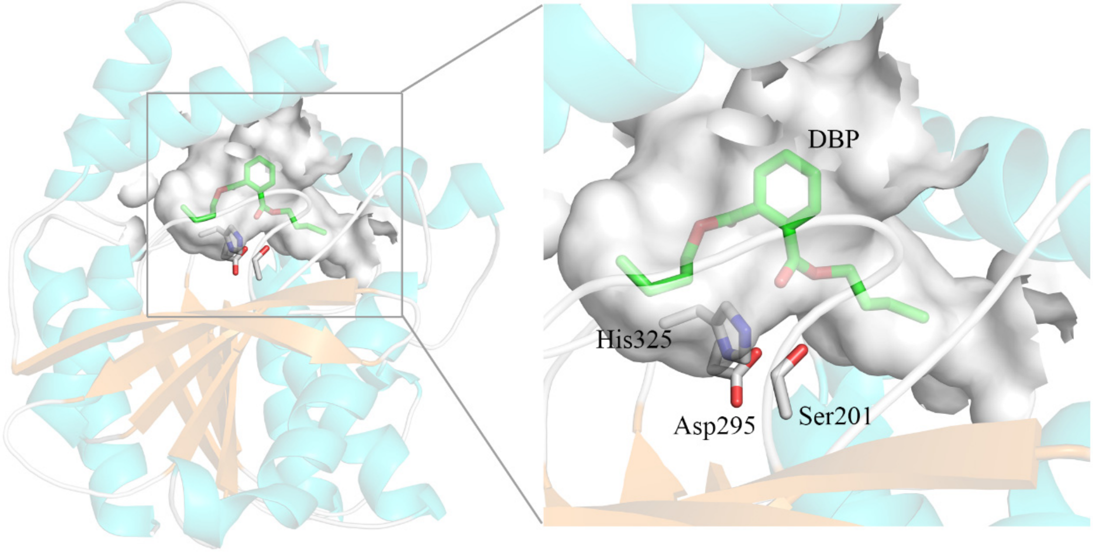

Figure S10. The SDS-PAGE analysis of hydrolase DphAN1 and its mutants. M: protein marker, 1: DphAN1, 2: S201A, 3: D295A, 4: H325A, 5: G129A, 6: G130A, 7: F78A, 8: V133A, 9: V230A, 10: D253A, 11: V254A, 12: V257A, 13: F330A.

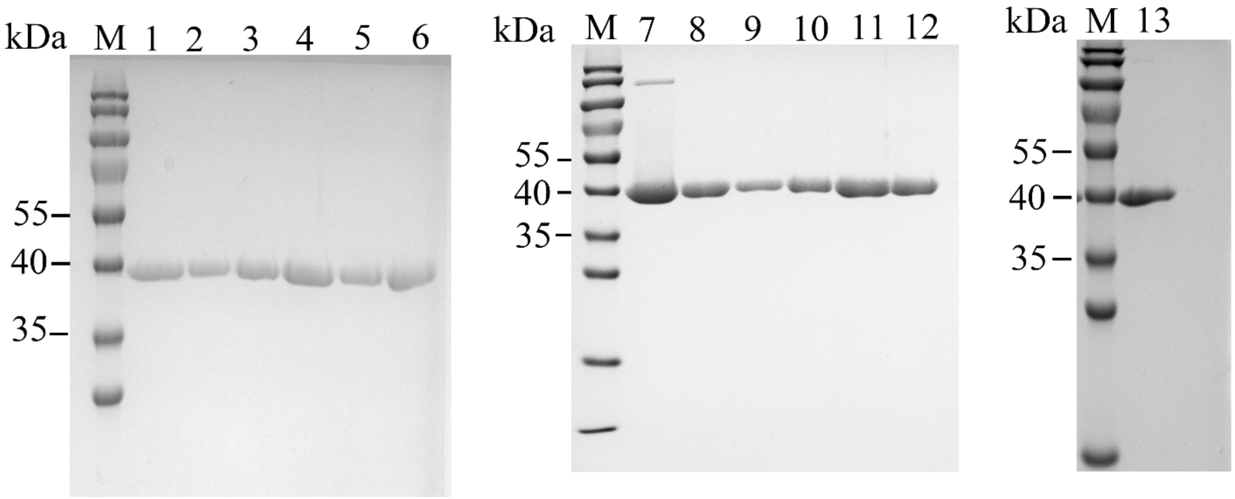

Supplement: Supplementary file 1 [file molecules-28-06738-s001.zip › molecules-2574942-supplementary.pdf]
